# Identification of a Potential Inhibitor (MCULE-8777613195-0-12) of New Delhi Metallo-β-Lactamase-1 (NDM-1) Using In Silico and In Vitro Approaches

**DOI:** 10.3390/molecules27185930

**Published:** 2022-09-13

**Authors:** Ghazala Muteeb, Md Tabish Rehman, Mohamed F. AlAjmi, Mohammad Aatif, Mohd Farhan, Sheeba Shafi

**Affiliations:** 1 Department of Nursing, College of Applied Medical Science, King Faisal University, Al-Ahsa 31982, Saudi Arabia; 2 Department of Pharmacognosy, College of Pharmacy, King Saud University, Riyadh 11451, Saudi Arabia; 3 Department of Public Health, College of Applied Medical Science, King Faisal University, Al-Ahsa 31982, Saudi Arabia; 4 Department of Basic Sciences, King Faisal University, Al-Ahsa 31982, Saudi Arabia

**Keywords:** antibiotic resistance, structure-based drug design, molecular docking, simulation, MCULE database, metallo-β-lactamase, steady-state enzyme kinetics

## Abstract

New Delhi metallo-β-lactamase-1 (NDM-1), expressed in different Gram-negative bacteria, is a versatile enzyme capable of hydrolyzing β-lactam rings containing antibiotics such as penicillins, cephalosporins, and even carbapenems. Multidrug resistance in bacteria mediated by NDM-1 is an emerging threat to the public health, with an enormous economic burden. There is a scarcity in the availability of specific NDM-1 inhibitors, and also a lag in the development of new inhibitors in pharmaceutical industries. In order to identify novel inhibitors of NDM-1, we screened a library of more than 20 million compounds, available at the MCULE purchasable database. Virtual screening led to the identification of six potential inhibitors, namely, MCULE-1996250788-0-2, MCULE-8777613195-0-12, MCULE-2896881895-0-14, MCULE-5843881524-0-3, MCULE-4937132985-0-1, and MCULE-7157846117-0-1. Furthermore, analyses by molecular docking and ADME properties showed that MCULE-8777613195-0-12 was the most suitable inhibitor against NDM-1. An analysis of the binding pose revealed that MCULE-8777613195-0-12 formed four hydrogen bonds with the catalytic residues of NDM-1 (His120, His122, His189, and Cys208) and interacted with other key residues. Molecular dynamics simulation and principal component analysis confirmed the stability of the NDM-1 and MCULE-8777613195-0-12 complex. The in vitro enzyme kinetics showed that the catalytic efficiency (i.e., *k*_cat_/*K*_m_) of NDM-1 on various antibiotics decreased significantly in the presence of MCULE-8777613195-0-12, due to poor catalytic proficiency (*k*_cat_) and affinity (*K*_m_). The IC_50_ value of MCULE-8777613195-0-12 (54.2 µM) was comparable to that of a known inhibitor, i.e., D-captopril (10.3 µM). In sum, MCULE-8777613195-0-12 may serve as a scaffold to further design/develop more potent inhibitors of NDM-1 and other β-lactamases.

## 1. Introduction

The emergence of multidrug-resistant bacterial species not only in hospitals but also in community settings poses a serious threat [1,2,3]. Such bacteria utilize various mechanisms to avoid the adverse effects of antibiotics/drugs. Some of the antibiotic resistant mechanisms include (i) the expression of β-lactamases capable of hydrolyzing β-lactam antibiotics, (ii) modification of the drug target, (iii) expression of drug efflux pumps, and (iv) alternate biosynthetic pathways. The production of β-lactamases is the most prevalent form of resistance in Gram-negative bacteria [4]. Ambler classifies β-lactamases into four classes (class A–D) based upon protein homology and their molecular mechanism of action. β-lactamases of class A, C, and D have serine residue at the catalytic center, and thus are known as serine β-lactamases [5]. Conversely, class B β-lactamases contain a metal ion at the catalytic center, and hence are called metallo-β-lactamases (MBLs) [6]. Further, Bush and Jacoby categorized β-lactamases into three groups (group 1–3) based on their functionality. Group 1 comprises cephalosporinases, while group 2 includes oxacillinases, penicillinases, extended-spectrum β-lactamases (ESBLs), and serine-based carbapenemases. The metal-based carbapenemases are classified into Group 3.

New Delhi metallo-β-lactamase-1 (NDM-1) is a versatile enzyme capable of hydrolyzing almost all types of antibiotics, such as penicillins, cephalosporins, and carbapenems [7]. NDM-1 is capable of cleaving the amide bond of β-lactam antibiotics, and thereby renders them ineffective. It is classified as Ambler’s Class B1, and Bush’s Group 3a β-lactamase. NDM-1 was first identified in India in a Swedish patient who was suffering from a *Klebsiella pneumoniae* urinary tract infection [8]. Afterwards, NDM-1 or its variants were reported throughout the world in Gram-negative bacteria such as *Escherichia coli*, *Acinetobacter baumanii*, *K. pneumoniae*, and *Enterobacter cloacae* [9]. Many inhibitors based on captoprils, natural compounds, thiols, Boric acid derivatives, and sulfinamides have been reported to show NDM-1 inhibition activity [10,11,12,13]. However, clinical usage of any of the putative inhibitors has not been approved. Therefore, in order to protect the existing β-lactam antibiotics, we need to design, discover, and/or develop new inhibitors of β-lactamases.

In this study, we screened a purchasable library of the MCULE database for potential inhibitors of NDM-1. We employed different computational approaches such as virtual screening, molecular docking, molecular dynamics simulation, and principal component analysis. Moreover, in vitro enzyme kinetics and IC_50_ determination validated the potential of the identified inhibitors.

## 2. Results 

### 2.1. Analysis of Binding Site by CASTp 3.0

The examination of NDM-1 and the hydrolyzed meropenem complex (PDB ID: 4EYL) by the CASTp 3.0 server identified only one binding site in NDM-1. The surface area and volume of the binding site were computed as 97.570 Å^2^ and 109.784 Å^3^, respectively. The amino acid residues that line the binding site were leu65, Phe70, Val73, Trp93, His120, His122, Glu123, Asp124, His189, Cys208, Lys211, Asn220, and His250.

### 2.2. Analysis of High-Throughput Virtual Screening

**Figure 1:**
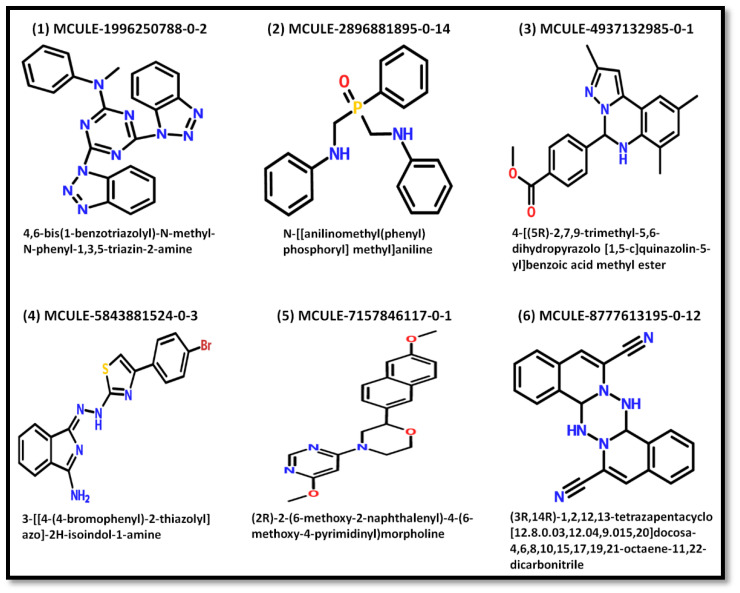
The 2D structures and chemical names of the shortlisted ligands obtained after virtual screening.

In a structure-based drug designing approach, a large database of small molecules was explored for their potential to bind with a target protein [14,15]. In the present study, 20,202,562 ligands in MCULE purchasable library were screened against NDM-1. The top 100 ligands obtained from the virtual screening were filtered by VINA score (Table 1). Only the ligands with a VINA score of ≤−8.0 kcal/mol (i.e., six ligands in total) were selected for further analysis. These shortlisted ligands were MCULE-1996250788-0-2, MCULE-8777613195-0-12, MCULE-2896881895-0-14, MCULE-5843881524-0-3, MCULE-4937132985-0-1, and MCULE-7157846117-0-1 (Figure 1). In order to confirm the high affinity between these six ligands and NDM-1, we again performed molecular docking using AutoDock 4.2. 

### 2.3. Validation of Docking Protocol 

The legitimacy of the docking procedure was confirmed by re-docking the ligand (i.e., hydrolyzed meropenem) found in the crystal structure of NDM-1, and comparing the RMSD between the two poses (Supplementary Figure S1). The RMSD between the docked pose and the crystal structure pose was 1.4568 Å, which indicated that the re-docked ligand occupied a similar binding pocket and formed native interactions with NDM-1. An RMSD value of less than 2.0 Å is considered to be acceptable for the molecular docking procedure [16]. Thus, the adopted docking protocol was accurate for the performed molecular docking between the selected ligands and NDM-1.

### 2.4. Analysis of Molecular Docking

#### 2.4.1. NDM-1 and Meropenem Interaction

**Figure 2:**
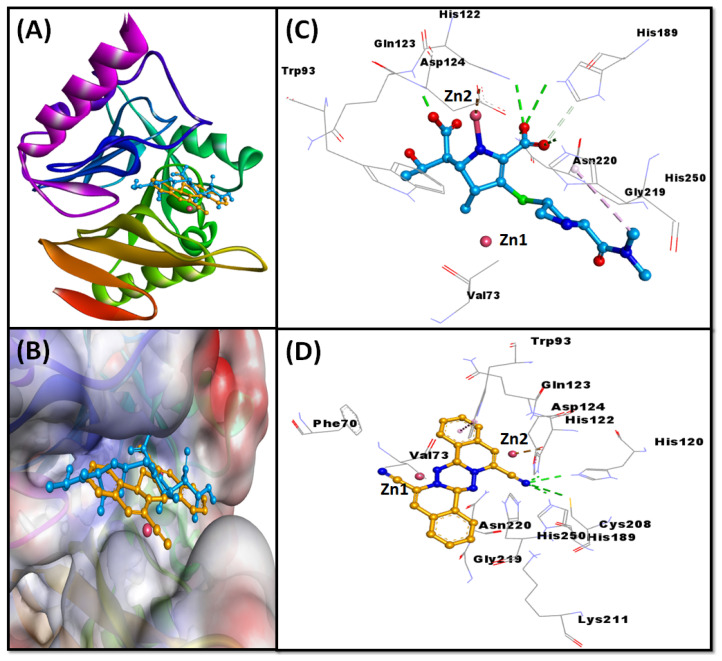
Molecular docking of NDM-1 with meropenem and MCULE-8777613195-0-12. (**A**) Binding of meropenem to the substrate binding site of NDM-1; (**B**) 3D representation of the interaction between NDM-1 and meropenem; (**C**) binding of MCULE-8777613195-0-12 to the substrate binding site of NDM-1; (**D**) 3D representation of the interaction between NDM-1 and MCULE-8777613195-0-12.

Molecular docking between the meropenem and NDM-1 suggested that it was bound to the substrate binding site of NDM-1 (Figure 2A). An investigation into the meropenem–NDM-1 interaction revealed that hydrogen bonding, hydrophobic, and electrostatic interactions stabilized the meropenem–NDM-1 complex. Meropenem formed seven hydrogen bonds with Asp124, His 122, Gln123, His189 (two interactions), Asn220, and His250. In addition, it interacted electrostatically with Asp124, and formed one hydrophobic interaction with His250 (Table 2). In addition, Val73, Trp93, and Gly219 of NDM-1 were engaged in van der Waals interactions with the meropenem (Figure 2B). The docking energy of the interaction between the meropenem and NDM-1 was estimated as −7.2 kcal mol^−1^.

#### 2.4.2. NDM-1 and MCULE-8777613195-0-12 Interaction

The molecular docking between MCULE-8777613195-0-12 and NDM-1 revealed that it occupied the substrate binding site of NDM-1 (Figure 2C). An analysis of the interaction between MCULE-8777613195-0-12 and NDM-1 advised that the hydrogen bonding primarily stabilized the protein–drug complex, with a small contribution from hydrophobic interactions. MCULE-8777613195-0-12 formed four hydrogen bonds with His120, His122, His189, and Cys208 (Figure 2D). A hydrophobic interaction was also formed with Trp93 (Table 2). Moreover, some residues of NDM-1 such as Zn1, Zn2, Phe70, Val73, Gln123, Asp124, Lys211, Gly219, Asn220, and His250 showed van der Waals interactions with MCULE-8777613195-0-12 (Figure 2D). The docking energy of the interaction between MCULE-8777613195-0-12 and NDM-1 was estimated as −8.9 kcal mol^−1^.

#### 2.4.3. NDM-1 and MCULE-1996250788-0-2 Interaction

Molecular docking between MCULE-1996250788-0-2 and NDM-1 suggested that it was bound to the substrate binding site of NDM-1 (Supplementary Figure S2A). The interaction between MCULE-1996250788-0-2 and NDM-1 was stabilized by hydrogen bonding, hydrophobic, and electrostatic interactions. MCULE-1996250788-0-2 formed two hydrogen bonds with His122 and Asn220. In addition, it interacted electrostatically with Val73, and showed two hydrophobic interactions with Zn1 and His250 (Table 2). In addition, Leu65, Phe70, His120, Gln123, Glu152, Met154, His189, and Lys211 of NDM-1 were engaged in van der Waals interactions with MCULE-1996250788-0-2 (Supplementary Figure S2B). The docking energy of the interaction between MCULE-1996250788-0-2 and NDM-1 was estimated as −8.4 kcal mol^−1^.

#### 2.4.4. NDM-1 and MCULE-2896881895-0-14 Interaction

The molecular docking between MCULE-2896881895-0-14 and NDM-1 revealed that it occupied the substrate binding site of NDM-1 (Supplementary Figure S2A). An analysis of the protein–drug complex of MCULE-2896881895-0-14 and NDM-1 interaction revealed that it was stabilized by a hydrogen bond, in addition to hydrophobic and electrostatic interactions. MCULE-2896881895-0-14 formed one hydrogen bond with Asp124, and showed two hydrophobic interactions with His122 and His250 (Table 2). In addition, some residues of NDM-1 such as Leu65, Val73, Trp93, His120, Gln123, His189, Lys211, Gly219, and Asn220 formed van der Waals interactions with MCULE-2896881895-0-14 (Supplementary Figure S2C). The docking energy of the interaction between MCULE-2896881895-0-14 and NDM-1 was estimated as −6.2 kcal mol^−1^.

#### 2.4.5. NDM-1 and MCULE-4937132985-0-1 Interaction

Molecular docking between MCULE-4937132985-0-1 and NDM-1 suggested that it was bound to the substrate binding site of NDM-1 (Supplementary Figure S2A). An investigation into the interaction between MCULE-4937132985-0-1 and NDM-1 revealed that the complex was stabilized by hydrogen bonding, in addition to hydrophobic and electrostatic interactions. MCULE-4937132985-0-1 formed two hydrogen bonds with Lys211 and His250. It also interacted electrostatically with His250, and formed seven hydrophobic interactions with Val73, Trp93 (two interactions), His122, His189, and His250 (two interactions) (Table 2). In addition, His120, Gln123, Asp124, Asp212, Ser217, Gly219, Asn220, and Ser251 of NDM-1 were engaged in van der Waals interactions with MCULE-4937132985-0-1 (Supplementary Figure S2D). The docking energy of the interaction between MCULE-4937132985-0-1 and NDM-1 was estimated as −8.0 kcal mol^−1^.

#### 2.4.6. NDM-1 and MCULE-5843881524-0-3 Interaction

The molecular docking between MCULE-5843881524-0-3 and NDM-1 revealed that it occupied the substrate binding site of NDM-1 (Supplementary Figure S2A). An analysis of the interaction between MCULE-5843881524-0-3 and NDM-1 revealed that the protein–drug complex was stabilized by hydrogen bond, hydrophobic, and electrostatic interactions. MCULE-5843881524-0-3 formed one hydrogen bond with His250, and two electrostatic interactions with His250 (Table 2). Moreover, it also formed seven hydrophobic interactions with Leu65, Val73, Trp93, Lys211, and His250 (three interactions). Moreover, some residues of NDM-1 such as Phe70, Ser217, Gly219, and Asn220 formed van der Waals interactions with MCULE-5843881524-0-3 (Supplementary Figure S2E). The docking energy of the interaction between MCULE-5843881524-0-3 and NDM-1 was estimated as −6.9 kcal mol^−1^.

#### 2.4.7. NDM-1 and MCULE-7157846117-0-1 Interaction

Molecular docking between MCULE-7157846117-0-1 and NDM-1 suggested that it was bound to the substrate binding site of NDM-1 (Supplementary Figure S2A). The MCULE-7157846117-0-1 and NDM-1 interaction complex was stabilized by hydrogen bonding and hydrophobic interactions. MCULE-7157846117-0-1 formed one hydrogen bond with Gln123. It also formed five hydrophobic interactions with Leu65, Val73, Trp93 (two interactions), and His250 (Table 2). In addition, Met67, His189, Lys211, Gly219, and Asn220 of NDM-1 were engaged in van der Waals interactions with MCULE-7157846117-0-1 (Supplementary Figure S2F). The docking energy of the interaction between MCULE-7157846117-0-1 and NDM-1 was estimated as −7.1 kcal mol^−1^.

### 2.5. Analysis of ADME Properties

The physicochemical and ADME properties of the shortlisted ligands (MCULE-1996250788-0-2, MCULE-8777613195-0-12, MCULE-2896881895-0-14, MCULE-5843881524-0-3, MCULE-4937132985-0-1, and MCULE-7157846117-0-1) were obtained using SwissADME portal (Table 3). The molecular weight of all the selected ligands was in the range of 336.37–420.43 g/mol; the number of rotatable bonds as 0–7; the numbers of hydrogen bond donors and acceptors were in 0–2 and 1–7, respectively. In addition, the molar refractivity and total polar surface area were in the ranges of 102.74–119.12 and 50.94–103.90 Å^2^, respectively (Table 3). Similarly, the lipophilicity of each selected ligand was evaluated using different algorithms, such as iLogP, xLogP3, wLogP, mLogP, and Silicos-IT. The consensus Log P_o/w_ of MCULE-1996250788-0-2, MCULE-8777613195-0-12, MCULE-2896881895-0-14, MCULE-5843881524-0-3, MCULE-4937132985-0-1, and MCULE-7157846117-0-1 was 3.21, 1.86, 3.64, 3.73, 3.74 and 2.77, respectively. All the ligands were moderately soluble as computed by the Log S (ESOL) method. Furthermore, all the shortlisted ligands possessed a high GI absorption property, and none of them were the substrate of P-gp except MCULE-1996250788-0-2 and MCULE-8777613195-0-12. 

Furthermore, the druglikeness of the shortlisted ligands was determined by computing its Lipinskis (Pfizer, New York, NY, USA), Ghose, Veber (GSK, London, UK), Egan (Pharmacia, Uppsala, Sweden), Muegge (Bayer, Leverkusen, German) and bioavialablity scores. We found that all the shortlisted ligands followed all the above stated rules except MCULE-1996250788-0-2, in which case Lipinski’s rule of five was violated (Table 3). The bioavailability score of all the selected ligands was 0.55. In terms of medicinal chemistry, there were no PAINS alerts in all the shortlisted ligands, and no Brenk alterations, except in MCULE-2896881895-0-14 and MCULE-5843881524-0-3. The overall synthetic accessibility of all the shortlisted ligands was in the range of 3.29–4.35. Finally, in terms of leadlikeness, only MCULE-8777613195-0-12 showed zero violations, while other shortlisted ligands showed one or more violations. Thus, on the basis of the ADME properties and molecular docking analysis, the ligand MCULE-8777613195-0-12 was selected as the best molecule which may exhibit the potential to inhibit NDM-1. Therefore, MCULE-8777613195-0-12 was subjected to molecular dynamics simulation and enzyme kinetics studies.

### 2.6. Analysis of MD Simulation

#### 2.6.1. Root Mean Square Deviation (RMSD)

**Figure 3:**
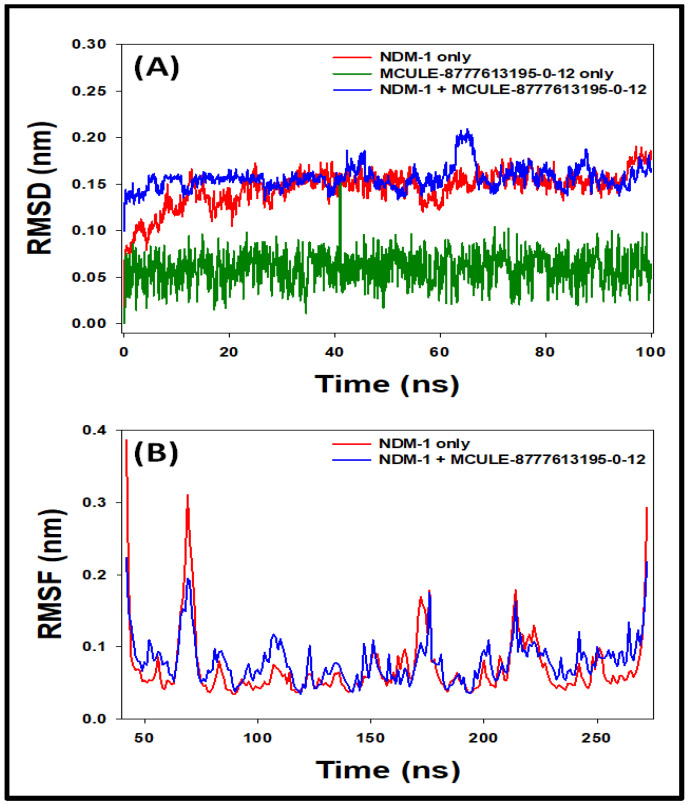
Molecular dynamics (MD) simulation of NDM-1 in the absence and presence of MCULE-8777613195-0-12. (**A**) Root mean square deviation (RMSD) in the Cα-atoms of NDM-1; (**B**) root mean square fluctuation (RMSF) in the side chains of NDM-1.

The RMSD is measured as a deviation in the protein—ligand complex structure from its initial state [17]. Figure 3A shows the RMSD values of NDM-1 in the absence and presence of MCULE-8777613195-0-12 as a function of simulation time. The RMSD values of NDM-1 alone fluctuated significantly during the first 10 ns and then became stable for the rest of simulation. The RMSD values of NDM-1 alone, MCULE-8777613195-0-12 alone, and the MCULE-8777613195-0-12 and NDM-1 complex were fluctuating in the ranges of 0.122–0.157 nm, 0.003–009 nm, and 0.146-0.201 nm, respectively. Since the RMSD values were within the acceptable range of 0.2 nm, this suggested that the structure of the protein alone or in complex form did not deviate significantly during the MD simulation [18]. The average RMSDs of NDM-1 alone, MCULE-8777613195-0-12 alone, and the MCULE-8777613195-0-12 and NDM-1 complex were 0.148 ± 0.011 nm, 0.006 ± 0.002 nm, and 0.157 ± 0.018 nm, respectively. The formation of a stable complex between NDM-1 and MCULE-8777613195-0-12 is clearly indicated by these results.

#### 2.6.2. Root Mean Square Fluctuation (RMSF)

The local conformational changes in the side chain of protein are generally measured by measuring RMSF [19]. The RMSF of NDM-1 in the absence and presence of MCULE-8777613195-0-12 was measured and the result is presented in Figure 3B. At the N- and C-terminal ends, the RMSF values were higher due to the higher flexibility of terminals. Considerably higher RMSF values were shown by some amino acid residues of NDM-1, which might be due to the entry or binding of MCULE-8777613195-0-12 at the substrate binding site of NDM-1.

#### 2.6.3. Radius of Gyration (Rg)

**Figure 4:**
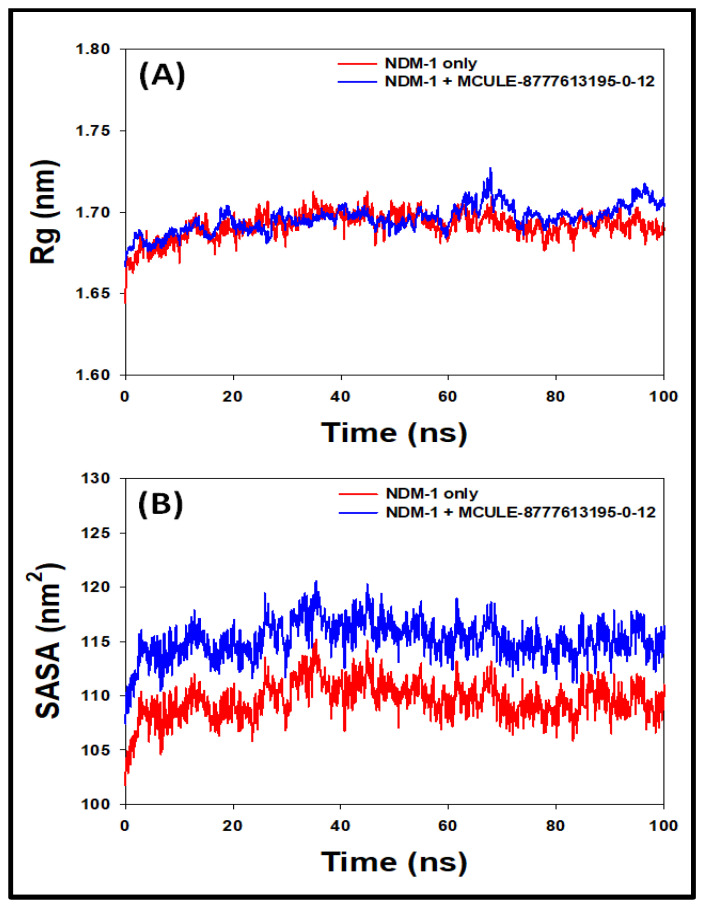
Variation in the (**A**) radius of gyration (rGyr); (**B**) solvent accessible surface area (SASA) of NDM-1 in the presence of MCULE-8777613195-0-12.

During MD simulation, the overall structure and folding state of a protein may be affected due to the binding of ligand. This can be easily measured by determining Rg as a function of simulation time [20]. Figure 4A depicts the variation in the Rg of NDM-1 in the absence and presence of MCULE-8777613195-0-12 during simulation. The Rg of NDM-1 alone during 20–100 ns fluctuated in the range of 1.67–1.71 nm, while the Rg of the NDM-1 and MCULE-8777613195-0-12 complex during 20–100 ns varied in the range of 1.68–1.73 nm. The fluctuations in both the cases were not significant and were within the acceptable limits. The average Rg values of NDM-1 alone and the NDM-1-MCULE-8777613195-0-12 complex during 20–100 ns were 1.69 ± 0.11 nm and 1.71 ± 0.14 nm, respectively. Clearly, the overall fluctuation in the Rg value of NDM-1 in the presence of MCULE-8777613195-0-12 was not significant, suggesting the stable nature of the protein–ligand complex.

#### 2.6.4. Solvent Accessible Surface Area (SASA)

The exposure of a protein–ligand complex to its surrounding solvent can be measured by calculating the SASA. It also signifies the overall packing of a protein–ligand system and its stability during MD simulation [17]. Figure 4B depicts the behavior of the SASA during MD simulation of NDM-1 in the absence and presence of MCULE-8777613195-0-12. Some minor fluctuations in the SASA of both systems were observed; however, they remained within the acceptable limits. The SASA values of NDM-1 alone or in the presence of MCULE-8777613195-0-12 during 20–100 ns were in the range of 106–114 nm^2^ and 111–120 nm^2^, respectively. The average SASA values of NDM-1 alone and the NDM-1–MCULE-8777613195-0-12 complex during 20–100 ns were 111 ± 12 nm^2^, and 117 ± 16 nm^2^, respectively. In brief, the formation of a stable NDM-1 and MCULE-8777613195-0-12 complex was suggested by the results of the SASA analysis, along with Rg.

### 2.7. Principal Component Analysis (PCA)

**Figure 5:**
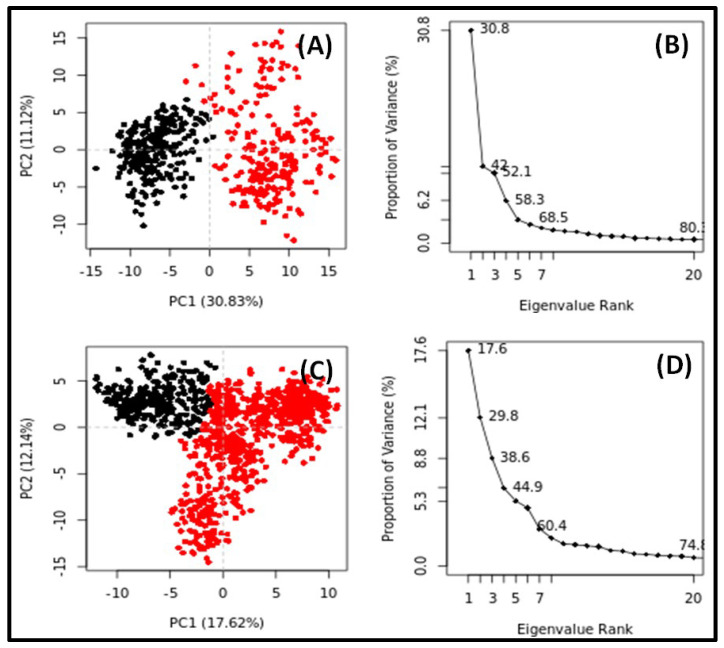
Principal component analysis (PCA) of NDM-1 in the absence and presence of MCULE-8777613195-0-12. (**A**) NDM-1 alone; (**B**) Variation in the variance (%) of NDM-1 alone as a function of ranked Eigenvalues; (**C**) NDM-1-MCULE-8777613195-0-12 complex; (**D**) Variation in the variance (%) of NDM-1 and MCULE-8777613195-0-12 complex as a function of ranked Eigenvalues.

PCA is a widely used method to examine the global motion of target proteins in the absence and presence of their respective ligands during simulation [21]. The conformational sampling of NDM-1 alone or in the presence of MCULE-8777613195-0-12 was computed along the PC1 and PC2 projected by the Cα-atoms (Figure 5). Each red and black dot represented a conformational state of NDM-1, while the red and black clusters indicate the presence of distinct energetically favorable conformational space. The conformational subspace occupied by NDM-1 alone spans from −15 to +15 along PC1 (30.83%), and −12 to +15 along PC2 (11.12%) (Figure 5A). It is noticeable that the first three eigenvalues of NDM-1 alone occupied 52.1% of the conformational variances (Figure 5B). Similarly, the conformational subspace occupied by NDM-1 in the presence of MCULE-8777613195-0-12 spans from −15 to +12 along PC1 (17.62%), and −15 to +10 along PC2 (12.14%) (Figure 5C). The first three eigenvalues of NDM-1 in the presence of MCULE-8777613195-0-12 occupied 38.6% of the conformational variances (Figure 5D). These results indicate that there is marginal decrease in the flexibility of NDM-1 in the presence of MCULE-8777613195-0-12, suggesting the formation of a stable protein–ligand complex. 

### 2.8. Analysis of Enzyme Kinetics Parameters

Steady-state enzyme kinetics was performed to evaluate the potential of the identified ligand, i.e., MCULE-8777613195-0-12, to inhibit NDM-1 enzyme activity. The NDM-1 enzyme alone was found to hydrolyze different β-lactam rings containing substrates such as ampicillin, cefotaxime, imipenem, and meropenem, in addition to a chromogenic substrate nitrocefin (Table 4). The affinity (*K*_m_; defined as the concentration of substrate at which the enzyme attains 50% of its maximum velocity), activity (*k*_cat_), and efficiency (*k*_cat_/*K*_m_) of NDM-1 against different substrates in the absence of MCULE-8777613195-0-12 were estimated to be in the ranges of 27.1–99.4 µM, 271.2–700.3 s^−1^, and 3.94–10.01 µM^−1^ s^−1^, respectively (Table 4). These results of NDM-1 kinetics in the absence of any inhibitor agreed with our previously published reports [22,23,24,25]. However, in the presence of MCULE-8777613195-0-12, the *K*_m_ of NDM-1 against different substrates was increased 1.0- to 2.0-fold, the *k*_cat_ values were decreased by 2.2 to 4.5 times, and the catalytic efficiency (*k*_cat_/*K*_m_) were decreased 2.5- to 6.6-fold (Table 4). For comparison, we also determined the kinetic parameters of NDM-1 in the presence of a known inhibitor, namely, D-captopril, using nitrocefin as substrate. The *K*_m_, *k*_cat_, and *k*_cat_/*K*_m_ of NDM-1 in the presence of D-captopril were estimated to be 78.6 ± 4.4 µM, 162.8 ± 16.3 s^−1^, and 2.07 ± 0.15 µM^−1^ s^−1^, respectively. It should be noted that the catalytic efficiency of NDM-1 pre-incubated with MCULE-8777613195-0-12 was comparable to that of D-captopril. These in vitro enzyme kinetics results proved that MCULE-8777613195-0-12 was a potent inhibitor of the NDM-1 enzyme. Since both *K*_m_ and *k*_cat_ values of NDM-1 were affected in the presence of MCULE-8777613195-0-12, a mixed kind of inhibition is anticipated.

### 2.9. Analysis of IC_50_ Value

**Figure 6:**
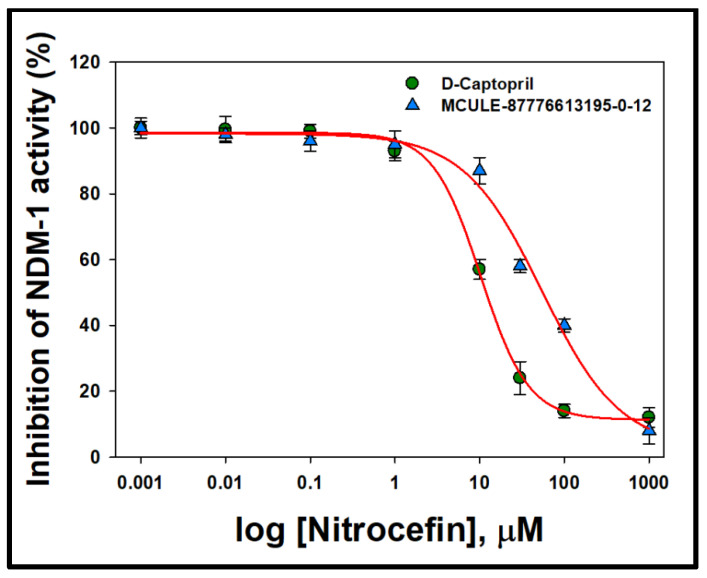
The IC_50_ curves of NDM-1 in the presence of D-captopril (known inhibitor) and MCULE-8777613195-0-12.

The potential of MCULE-87776613195-0-12 to inhibit NDM-1 was accessed by determining IC_50_ value and comparing it with that of a known NDM-1 inhibitor, i.e., D-captropril (Figure 6). The IC_50_ values of MCULE-87776613195-0-12 and D-captopril were estimated as 54.2 ± 6.3 µM and 10.3 ± 2.7 µM, respectively. Earlier, the IC_50_ values of D-captopril were reported to be 7.9–11.8 µM [25,26], which was close to the value obtained in this study. Since the IC_50_ value of MCULE-87776613195-0-12 was around 5-fold higher than the known inhibitor (D-captopril), the potential of the identified drug molecule as a potent inhibitor of NDM-1 is revealed.

## 3. Discussion

Antibiotic resistance in bacteria and its global spread is an arising hazard to human health with great commercial consequences. Despite the severity of the situation, no clinical inhibitors are available against β-lactamase expressing bacteria, which is considered to be the main reason for antibiotic resistance [27,28]. Among the β-lactamases, metallo-β-lactamases such as NDM-1 are the most potent and versatile enzymes, capable of cleaving nearly all the feasible antibiotics, including carbapenems. Thus, NDM-1 is the most appropriate target in which to identify novel inhibitors in order to contain the spread of antibiotic resistance in bacteria [28,29]. Earlier studies also suggest the suitability of NDM-1 as the most promising drug target to identify inhibitors such as ethylenediamine derivatives [30,31], pyridine derivatives [32], spiro-indole-thiadiazole derivatives [33], magnolol derivatives [34], pterostilbenes [35], sulfur-containing carboxylic acids [10,11], dithioazolidine derivatives [36], dipicolinic acids [37], phosphates [38], cyclic borates [7], Bi(III) compounds [39], chromones [40], sulfonamides [12], triazothioacetamides [41], and natural compounds [13]. This motivated us to screen a large database (MCULE’s purchasable database, containing more than 20 million compounds) to discover novel non-β-lactam ring encompassing inhibitors against NDM-1. The existing mechanism of defense in resistant bacteria enables them to hydrolyze the β-lactam ring containing antibiotics/inhibitors. Thus, non-β-lactam-based inhibitors would be a good choice against antibiotic resistant bacteria as such, inhibitors would not be inactivated and hydrolyzed by them [1]. In this article, multi-dimensional approaches such as virtual screening, molecular docking/dynamics, principal component analysis, and in vitro enzyme kinetics were applied to identify NDM-1 inhibitors.

The screening of MCULE’s database containing more than 20 million compounds by AutoDock Vina led to the identification of six molecules, namely, MCULE-1996250788-0-2, MCULE-8777613195-0-12, MCULE-2896881895-0-14, MCULE-5843881524-0-3, MCULE-4937132985-0-1, and MCULE-7157846117-0-1 as the most potent inhibitors of NDM-1. In order to confirm the binding of shortlisted ligands to NDM-1, we again performed molecular docking using AutoDock 4.2. Among the shortlisted ligands, MCULE-8777613195-0-12 displayed the lowest binding energy (−8.9 kcal/mol), followed by MCULE-1996250788-0-2 (−8.4 kcal/mol), MCULE-4937132985-0-1 (−8.0 kcal/mol), MCULE-7157846117-0-1 (−7.1 kcal/mol), MCULE-5843881524-0-3 (−6.9 kcal/mol), and MCULE-2896881895-0-14 (−6.2 kcal/mol). Furthermore, the suitability of the shortlisted ligands to be developed as potential drug molecules was confirmed by analyzing their ADME properties. The druglikeness of the candidate ligands was evaluated by various standard rules such as those of Lipinski (Pfizer), Ghose, Veber (GSK), Egan (Pharmacia), and Muegge (Bayer). Ghose et al. (1999) suggest that a candidate drug molecule should have a molecular weight, molar refractivity, total number of atoms, and log P should be in the range of 160–480 g/mol, 40–130, 20–70, and −0.4 to 5.6, respectively [42]. In another study, Veber and coworkers (2002) noted that the probability of a drug molecule to have good oral bioavailability depends on (i) 10 or fewer rotatable bonds, (ii) lower polar surface area (<140Å^2^), and (iii) 12 or fewer hydrogen bond donors and acceptors [43]. Keeping these filters in mind, all the ligands were found to pass, except MCULE-1996250788-0-2. Moreover, the medicinal chemistry properties of the candidate ligands were accessed by PAINS, Brenk, leadlikeness, and synthetic accessibility. All the ligands passed the PAINS test, while in the Brenks test, two ligands, namely, MCULE-2896881895-0-14 and MCULE-5843881524-0-3, showed one alert. Among all the shortlisted ligands, only MCULE-8777613195-0-12 showed leadlike properties, with a synthetic accessibility score of 4.35. Hence, MCULE-8777613195-0-12 was selected for further studies such as MD simulation, PCA, in vitro enzyme kinetics, and IC_50_ determination.

The X-ray crystal structure of NDM-1 revealed that NDM-1 has a αβ/βα conformation, with a deep central active site containing two Zn ions. The Zn1 is coordinated with His120, His122, and His189 in a tetrahedral geometry, while Zn2 is coordinated with Asp124, Cys208 and His250 in a trigonal pyramidal geometry [44,45]. These residues are significant in maintaining the proper orientation of di-Zn ions in the catalytic center [29]. An analysis of the docking pose of MCULE-8777613195-0-12 inside the catalytic site of NDM-1 indicates that MCULE-8777613195-0-12 interacted with NDM-1 through hydrogen bonding with key catalytic residues such as His120, His122, His189, and Cys208. In addition to the catalytic residues, some non-active residues also play significant role in maintaining the proper orientation of the substrate for feasible hydrolytic reaction. During substrate binding, Met67 moves away from the di-Zn center by ~4.9 Å. This re-orientation brings Leu65 closer to the di-Zn center by ~2.1 Å [29]. Trp93 along with Met67 and Leu65 facilitate the entry of substrate towards the active site. Moreover, as a result of these movements, Asn220 is pulled ~1.0 Å closer to the di-Zn center, where it interacts with the carbonyl group of the substrate. Consequently, an oxy-anion hole is created at the substrate by Asn220 and Zn1, thereby facilitating hydrophilic attack by hydroxide ion, which was produced from the water molecule attached to Asp124 [25]. We also noticed that MCULE-8777613195-0-12 and NDM-1 showed a hydrophobic interaction with Trp93, and van der Waals interactions with Zn1, Zn2, Phe70, Val73, Gln123, Asp124, Lys211, Gly219, Asn220, and His250. The van der Waals interactions play significant role in determining the formation of a stable protein–ligand complex. These are distance-dependent forces; most act collectively to make an impact. The models based on the Lennard–Jones potential are useful in accurately estimating van der Waals interactions and, thus, are useful in molecular docking simulations and the virtual screening of large databases. Furthermore, the stability of the NDM-1 and MCULE-8777613195-0-12 complex was probed by MD simulation; the results (RMSD, RMSF, Rg, and SASA) suggest the formation of a stable NDM-1 and MCULE-8777613195-0-12 complex. These results were also confirmed by PCA analysis. Furthermore, the effect of MCULE-8777613195-0-12 on NDM-1 activity was evaluated on a number of substrates such as ampicillin, cefotaxime, imipenem, and meropenem, in addition to a chromogenic substrate, nitrocefin. The results confirmed that the binding of MCULE-8777613195-0-12 to NDM-1 reduced its affinity towards substrates, as well as its activity. 

The IUPAC name of MCULE-8777613195-0-12 is [3R,14R]-1,2,12,13-tetrazapentacyclo [12.8.0.03,12.04,9.015,20]docosa-4,6,8,10,15,17,19,21-octaene-11,22-dicarbonitrile. Carbonitriles have been shown to possess anti-bacterial activity. Recently, Wockhard Ltd. (Aurangabad, India) reported the inhibitory potential of a nitrile salt equivalent to avibactam [46]. McGeary et al. (2017) synthesized a number of 2-aminopyrrole-1-benzyl-4,5-diphenyl-1H-pyrrole-3-carbonitrile derivatives and evaluated their potential as broad spectrum inhibitors of metallo-β-lactamases such as IMP-1 (B1 sub-group), CphA (B2 sub-group), and AIM-1 (B3 sub-group) [47]. Likewise, broad-spectrum anti-bacterial and anti-fungal activities of 6-phenyl-2,4-disubstituted pyrimidine-5-carbonitriles derivative were reported by Al-Abdullah et al. (2011) [48]. In sum, MCULE-8777613195-0-12 binds to the active site of NDM-1 and forms a stable complex. As the binding potential of MCULE-8777613195-0-12 towards NDM-1 is higher than for β-lactam antibiotics, it may contest with antibiotics to occupy the enzyme’s active site, and thereby allow them to survive the hydrolysis by NDM-1.

## 4. Materials and Methods

### 4.1. Materials

Ampicillin, cefotaxime, imipenem, meropenem, D-captopril, and 4-(2-pyridylazoresorcinaol (PAR) were purchased from Sigma (St. Louis, MO, USA). Chromogenic substrate analog nitrocefin was procured from Calbiochem (St. Louis, MO, USA). The inhibitor MCULE-87776613195-0-12 was bought from MCULE Inc. (Palo Alto, CA, USA).

### 4.2. Binding Site Determination Using CASTp3.0

The 3D structure of NDM-1 bound with hydrolyzed meropenem (control ligand) was submitted to CASTp3.0 server [49] to identify the most suitable binding site present on NDM-1.

### 4.3. Preparation of Ligands/Protein, and Virtual Screening

The MCULE purchasable (in stock) library was used for high-throughput virtual screening (accessed on 09/03/2022). The high-throughput virtual screening was performed using “MCULE online drug discovery platform”, as described previously [46]. The molecules in the library were filtered using the “Basic property filter” of the platform. Various properties such as components, inorganic atoms, rotatable bonds, chiral centers, RO5 violations, heavy atoms, N/O atom, rings, and halogen atoms were available to filter the molecules. The minimum and maximum values in the screening input parameters were defined based on the values of meropenem. A total of 20,202,562 ligands was selected for screening purposes against the NDM-1 active site. The values of sampler size and maximum number of compounds after sphere exclusion were set to 1000 and 3,000,000, respectively. The other options were set to their default values.

The 3D coordinates of the target protein (NDM-1) were downloaded from RCSB databank (PDB ID: 4EYL). The NDM-1 crystal structure with bound hydrolyzed meropenem was resolved to 1.90 Å [44]. Prior to molecular docking, protein was prepared by adding H-atoms, assigning bond orders, removing any heteroatoms including, and deleting all non-catalytic water molecules. The changes in Zn ions were maintained. A new hydrogen bond network was defined, and the energy of the system was minimized using the CHARMM36 force field. Virtual screening was performed using the AutoDock Vina-enabled MCULE screening platform. The pre-processed file of NDM-1 protein was uploaded to the MCULE screening platform. A grid box of 20.8 Å, 23.0 Å, and 24.2 Å dimensions, centered at 8.3 Å, −40.0 Å, and 6.2 Å, was used for the screening purpose. All the ligands were ranked by VINA score and the top six ligands with a VINA score of ≤ −8.0 kcal/mol were selected for further study.

### 4.4. Molecular Docking and Validation of Protocol

The molecular docking of the top-ranked ligands against NDM-1 was again performed using AutoDock4.2, as suggested earlier [50,51]. Briefly, the molecular docking was performed inside a grid box with dimensions of 20.8 Å, 23.0 Å, and 24.2 Å, centered at 8.3 Å, −40.0 Å, and 6.2 Å with a spacing between grid points of 0.375 Å. Lamarck Genetic Algorithm (LGA) was used for the global search while the Solis–Wets method was used for the local search of the binding site of ligands inside NDM-1’s active site. A total of 2.5 × 10^6^ energy calculations were performed for each docking run, and a total of 50 docking runs were computed. The values of population size, translational step, torsion steps, and quaternions were set to 150, 0.2 Å, 5, and 5, respectively. The molecular interaction between the ligand and protein was identified using Discovery Studio Visualizer 4.1. The dissociation constant (*K_d_*) for the NDM-1 and ligand interaction was calculated from the docking energy (Δ*G*) using the following relation, and as defined earlier [52,53]:

$$\Delta G=-RT\ln K_{d}$$

where *R* and *T* are the universal gas constant and temperature, respectively.

The validation of the molecular docking protocol was performed by re-docking the ligand (meropenem), which was bound to the NDM-1 active site in its crystal structure. For this purpose, the bound ligand was first extracted from the crystal structure and then docked again to the active site of NDM-1 using the same set of docking parameters. Finally, the root mean square deviation (RMSD) between the docked and crystal structure poses was computed by super-imposing the two structures. 

### 4.5. Determination of ADME Properties

The pharmaco-kinetic, i.e., adsorption, distribution, metabolism, and excretion (ADME), properties of the top six ligands were determined using SwissADME [54]. In addition, the druglikeness of the selected ligands was determined by performing several tests such as those of Lipinski, Ghose, Veber, Egan, PAINS, and Muegge. The top-rated ligand was identified which had favorable ADME properties and passed all the aforementioned tests for druglikeness. Lipinski’s rule of five states that a druglike molecule should have a molecular mass of less than 500 Da; it should have no more than 5 hydrogen bond donors and 10 hydrogen bond acceptors; and its lipophilicity (i.e., MlogP) should be less than 5. Similarly, the Ghose filter suggests a druglike molecule should have a molecular weight in the range of 160–480 Da, its lipiphilicity (WlogP) should be in the range of −0.4 to 5.6, with a molar refractivity ≤130, and the number of atoms should be in the range of 20–70 [42]. Likewise, Veber’s filter indicates the number of rotatable bonds should be ≤10 and the total polar surface area should be ≤140 [43]. Further, Egan’s filter shows that lipophilicity (WlogP) should be ≤5.88 and the total polar surface area should be ≤131. Furthermore, Muegge’s filter suggests that the molecular weight of a druglike molecule should be within the range of 200–600 Da, its lipophilicity (XlogP3) should be in the range of −2 to 5, its total polar surface area should be ≤150, the number of rings should be ≤7, the number of carbon should be >4, the number of heteroatoms should be >1, the number of rotatable bonds should be ≤15, the number of hydrogen bond acceptors should be ≤10, and the number of hydrogen bonds should be ≤5.

### 4.6. Molecular Dynamics (MD) Simulation

Molecular dynamics (MD) simulation was performed to evaluate the stability of the NDM–ligand complex using Gromacs 2020.4 installed on a workstation powered by Intel Xenon E3-1245 with eight cores, a 3.50 GHz processor, 32 GB RAM, and an NVIDIA Quadro P5000 GPU card [55,56]. The pdb2gmx command of GROMACS was used to generate protein topology using a CHARMM36-all atom forcefield, and TIP3P water molecules, while CHARMM General Force Field (CGenFF) was used to generate the ligand’s topology. A dodecahedron box was used to perform the MD simulation after placing the NDM-1 and ligand complex at the center, at least 1.0 nm away from the boundaries of the box. A total of 17,014 TIP3P water molecules were used to solvate the simulation box, and 5 Na+ ions were added to neutralize the system. Furthermore, NaCl (150 mM) was added to imitate the physiological conditions. A maximum 50,000 steps was used to minimize the energy of the system by the steepest descent minimization method. Furthermore, isothermal–isochroic (NVT) and isothermal–isobaric (NPT) ensembles were accomplished at a temperature of 300 K and a pressure of 1.0 bar, which were maintained throughout using a Berendsen thermostat and Parrinello–Rahman barostat. Finally, a production run of 100 ns was performed on the equilibrated system, with a time-step of 2 fs that was fixed using a leap-frog integrator. The algorithm for NVT, NPT, and the production runs was constrained using LINCS. Finally, the MD simulation results were investigated for root mean square deviation (RMSD), root mean square fluctuation (RMSF), radius of gyration (Rg), and solvent accessible surface area (SASA) [20,55]. All the experiments were performed independently in triplicates and the results are reported as mean ± standard errors. 

### 4.7. Principal Component Analysis (PCA)

Principal component analysis (PCA) or essential dynamics (ED) is a widely used method to compute the conformational flexibility of protein in the presence of ligands by measuring their collective motions. In this study, the PCA of NDM-1 in the presence of the top-rated ligand was performed using Bio3D, as reported previously [57,58]. In PCA, the translational and rotational motions of the protein were first removed. Then, the atomic coordinates’ positional covariance matrix and its eigenvectors were computed by superimposing the coordinates of the protein onto a reference structure. Later, a diagonal matrix of eigenvalues was generated by diagonalizing the calculated symmetric matrix by an orthogonal coordinate transformation matrix. In this matrix, each eigenvector represents an eigenvalue associated with the total mean–square fluctuation of the system, along the corresponding eigenvector. The covariance matric (*C*) is calculated using the following relation.

$$C_{ij}=\langle \left(x_{i}-\langle x_{i}\rangle \right)\left(x_{j}-\langle x_{j}\rangle \right)\rangle \;\;\;\;\;\left(i,j=1,2,3,\ldots .,3N\right)$$

where *N*, *x_i/j_*, and <*x_i/j_*> represent the number of Cα-atoms, the Cartesian coordinate of the ith/jth Cα-atom, and time average of all the conformations, respectively. 

### 4.8. Determination of Enzyme Kinetics Parameters

The purified NDM-1 was obtained from GenScript (New Jersey, NJ, USA) and its zinc content was determined using PAR (4-(2-Pyridylazo) resorcinol) assay, as described elsewhere [23]. The concentration of NDM-1 was determined spectrophotometrically using a molar extinction coefficient of 27,800 M^−1^ cm^−1^. The stock solution of inhibitor was prepared in DMSO and then diluted in the assay buffer (final DMSO concentration was less than 0.5%). Furthermore, the steady-state enzyme kinetics was performed to evaluate the hydrolytic activity of NDM-1 in the presence of the top-rated ligand, as reported previously [25]. The change in the molar extinction coefficients of the antibiotics/substrates upon hydrolysis were Δε_486_ = +15,000 M^−1^ cm^−1^ for nitrocefin, Δε_235_ = −900 M^−1^ cm^−1^ for ampicillin, Δε_264_ = −7,250 M^−1^ cm^−1^ for cefotaxime, Δε_295_ = −10,500 M^−1^ cm^−1^ for imipenem, and Δε_297_ = −10,940 M^−1^ cm^−1^ for meropenem. The enzymatic reaction was performed in 50 mM HEPES buffer (pH 7.0) containing NaCl (0.25 M), and ZnCl_2_ (0.1 mM) at 30 °C. BSA (with no hydrolytic activity of its own) was added at a. concentration of 20 µg/ml to the reaction buffer to prevent the denaturation of NDM-1. Different concentrations of antibiotics/substrates were incubated with varying concentrations (0.1 to 2 nM) of NDM-1. The initial velocities were calculated from the observed change in absorbance upon antibiotic/substrate hydrolysis and the kinetic parameters (*K*_m_ and *k*_cat_) were calculated using the Michaelis–Menten equations:

$$v=\frac{V_{max}\;\left[S\right]}{K_{m}+\left[S\right]}$$

$$k_{cat}=\frac{V_{max}}{\left[E\right]}$$

where *v*, *V_max_*, [*S*], and [*E*] are initial velocity, maximum velocity, substrate concentration, and enzyme concentration, respectively.

Three independent experiments were performed and the results are reported as mean ± standard error.

### 4.9. Determination of IC_50_

The IC_50_ value of a ligand/drug is defined as the concentration at which the activity of an enzyme is reduced by 50%. In this study, the IC_50_ values of the top-rated ligand and D-captopril (as control) were determined by observing the hydrolysis of 100 µM nitrocefin at 486 nm, as published earlier [26]. Briefly, the NDM-1 enzyme (0.5 nM) was incubated for 5 min at 30 °C with varying concentrations (0.001 to 1000 µM) of the ligand and D-captopril. The change in absorbance due to the hydrolysis of nitrocefin was converted into enzyme activity and a plot of activity versus log (ligand) concentration was plotted to compute the *IC*_50_ values, using the following relation in a Sigma plot:

$$Y=\frac{\left(max-min\right)}{1+10^{\left(\left(logIC_{50}-X\right)\times Hill\;slope\right)}}$$

where *X* represents inhibitor concentration, *Y* represents %inhibition data, *IC*_50_ is the concentration of substrate at which the activity is reduced by 50%, and Hill slope is the slope factor of the plot.

The results are reported as mean ± standard error of three independent experiments.

## 5. Conclusions

In this study, the MCULE purchasable library was screened against NDM-1 using AutoDock VINA to identify novel inhibitors. Based on VINA score (≤ −8.0 kcal/mol), six ligands (MCULE-1996250788-0-2, MCULE-8777613195-0-12, MCULE-2896881895-0-14, MCULE-5843881524-0-3, MCULE-4937132985-0-1, and MCULE-7157846117-0-1) were identified to bind the active site of NDM-1. The binding of the top-ranked ligands was confirmed by performing molecular docking using AutoDock4.2. Furthermore, the ADME profiling of the shortlisted ligands suggests that MCULE-8777613195-0-12 was the only ligand which satisfied all the pharmacokinetic as well as the druglikeness properties. Therefore, the interaction between MCULE-8777613195-0-12 and NDM-1 was further studied by analyzing the molecular docking interaction pattern through MD simulation and PCA. The analysis of the molecular docking pose confirmed that MCULE-8777613195-0-12 occupied the substrate binding site of NDM-1. Several key catalytic residues of NDM-1 such as His120, His122, His189, and Cys208 formed hydrogen bonds with MCULE-8777613195-0-12. In addition, Zn1 and Zn2 ions of NDM-1 interacted with the shortlisted ligand through van der Waals interaction. In addition, some essential but non-active site residues such as Phe70, Val73, Trp93, Asp124, Asn220 and His250 also interacted with NDM-1. Furthermore, MD simulation and PCA analysis confirmed the stability of the NDM-1 and MCULE-8777613195-0-12 complex. Furthermore, the potential of MCULE-8777613195-0-12 to inhibit NDM-1 activity was affirmed by in vitro enzyme kinetics. Hence, MCULE-8777613195-0-12 may serve as a promising seed molecule for the design of more potent NDM-1 inhibitors.

## Figures and Tables

**Table 1:** List of top 100 ligands identified by screening MCULE’s purchasable library using AutoDock Vina.

S. No.	MCULE ID	Vina Docking Score (kcal mol^−1^)	S. No.	MCULE ID	Vina Docking Score (kcal mol^−1^)
1.	MCULE-1996250788-0-2	−8.6	51.	MCULE-6386698026-0-1	−7.1
2.	MCULE-8777613195-0-12	−8.5	52.	MCULE-5341990674-0-1	−7.1
3.	MCULE-2896881895-0-14	−8.3	53.	MCULE-7413800685-0-1	−7.1
4.	MCULE-5843881524-0-3	−8.1	54.	MCULE-6611889679-0-1	−7.0
5.	MCULE-4937132985-0-1	−8.0	55.	MCULE-3757189842-0-3	−7.0
6.	MCULE-7157846117-0-1	−8.0	56.	MCULE-1449339079-0-1	−7.0
7.	MCULE-2703793620-0-19	−7.9	57.	MCULE-7198966831-0-5	−7.0
8.	MCULE-6435437822-0-4	−7.9	58.	MCULE-6102208381-0-1	−7.0
9.	MCULE-9087653650-0-1	−7.9	59.	MCULE-1487541190-0-1	−7.0
10.	MCULE-5502880115-0-1	−7.8	60.	MCULE-6333662959-0-3	−7.0
11.	MCULE-5902134438-0-2	−7.7	61.	MCULE-7923495923-0-1	−7.0
12.	MCULE-3249854796-0-1	−7.7	62.	MCULE-7835028598-0-4	−7.0
13.	MCULE-3846475833-0-1	−7.6	63.	MCULE-4126490714-0-1	−7.0
14.	MCULE-1919838085-0-4	−7.6	64.	MCULE-7851870038-0-1	−7.0
15.	MCULE-6788465213-0-1	−7.5	65.	MCULE-8821315043-0-1	−7.0
16.	MCULE-6852650200-0-1	−7.5	66.	MCULE-2698734795-0-2	−7.0
17.	MCULE-4913117548-0-1	−7.5	67.	MCULE-9350639909-0-11	−6.9
18.	MCULE-6421424274-0-1	−7.5	68.	MCULE-9183103932-0-1	−6.9
19.	MCULE-1186494303-0-1	−7.5	69.	MCULE-8087049601-0-5	−6.9
20.	MCULE-9342953905-0-2	−7.5	70.	MCULE-1791008433-0-8	−6.9
21.	MCULE-9631694698-0-5	−7.4	71.	MCULE-3466797615-0-2	−6.9
22.	MCULE-8059370584-1-1	−7.4	72.	MCULE-1317104543-0-7	−6.9
23.	MCULE-1062480345-0-1	−7.4	73.	MCULE-9378438314-0-42	−6.9
24.	MCULE-2810458334-0-1	−7.4	74.	MCULE-4617546830-0-3	−6.9
25.	MCULE-9072200022-0-8	−7.4	75.	MCULE-7017115425-0-3	−6.9
26.	MCULE-1898707422-0-1	−7.4	76.	MCULE-3727428710-0-1	−6.9
27.	MCULE-7966568047-0-1	−7.3	77.	MCULE-3328329188-0-2	−6.9
28.	MCULE-2911526489-0-1	−7.3	78.	MCULE-2331495250-0-1	−6.9
29.	MCULE-6532940525-0-24	−7.3	79.	MCULE-4377304507-0-2	−6.9
30.	MCULE-1939909394-0-16	−7.3	80.	MCULE-5948461200-0-1	−6.9
31.	MCULE-3431042829-0-4	−7.3	81.	MCULE-3275768312-0-3	−6.8
32.	MCULE-3078030187-0-1	−7.3	82.	MCULE-1135254215-0-1	−6.8
33.	MCULE-3731485659-0-1	−7.3	83.	MCULE-2772971417-0-1	−6.8
34.	MCULE-5531242712-0-4	−7.3	84.	MCULE-2133174213-0-55	−6.8
35.	MCULE-8263613910-0-1	−7.3	85.	MCULE-9413565617-0-1	−6.8
36.	MCULE-4331631183-0-3	−7.2	86.	MCULE-7782925588-0-1	−6.8
37.	MCULE-2906178381-0-1	−7.2	87.	MCULE-8599394063-0-2	−6.8
38.	MCULE-9300230391-0-8	−7.2	88.	MCULE-7335024771-0-1	−6.8
39.	MCULE-3251857628-0-1	−7.2	89.	MCULE-6762916380-0-3	−6.8
40.	MCULE-1167043622-0-2	−7.2	90.	MCULE-2195622093-0-1	−6.8
41.	MCULE-1936444400-0-4	−7.2	91.	MCULE-3973310327-0-5	−6.8
42.	MCULE-6861305276-0-1	−7.1	92.	MCULE-5557610945-0-1	−6.8
43.	MCULE-9426734282-0-11	−7.1	93.	MCULE-4164396300-0-1	−6.7
44.	MCULE-6177462090-0-1	−7.1	94..	MCULE-9583784629-0-2	−6.7
45.	MCULE-6227113621-0-37	−7.1	95.	MCULE-4090232208-0-4	−6.7
46.	MCULE-1811168249-0-7	−7.1	96.	MCULE-9229703054-0-1	−6.7
47.	MCULE-5864809920-0-1	−7.1	97.	MCULE-7386838022-0-3	−6.7
48.	MCULE-6461370294-0-1	−7.1	98.	MCULE-8236708587-0-1	−6.7
49.	MCULE-8281707653-0-1	−7.1	99.	MCULE-2439091863-0-1	−6.7
50.	MCULE-6173356820-0-1	−7.1	100.	MCULE-9011292933-0-1	−6.7

**Table 2:** Molecular docking parameters of shortlisted ligands and NDM-1 interaction.

Name of Drug	Hydrogen Bonding	Hydrophobic Interactions	Electrostatic Interactions	van der Waals Interactions	AutoDock 4.2 Score (kcal/mol)
Meropenem (control)	Asp124, His122, Gln123, His189*, Asn220, His250	His250	Asp124	Val73, Trp93, Gly219	−7.2
MCULE-1996250788-0-2	His122, Asn220	Val73	Zn1, His250	Leu65, Phe70, His120, Gln123, Glu152, Met154, His189, Lys211	−8.4
MCULE-2896881895-0-14	Asp124	His122, His250	Zn1, Asp124, His250 *	Leu65, Val73, Trp93, His120, Gln123, His189, Lys211, Gly219, Asn220	−6.2
MCULE-4937132985-0-1	Lys211, His250	Val73, Trp93 *, His112, His189, His250 *	His250	His120, Gln123, Asp124, Asp212, Ser217, Gly219, Asn220, Ser251	−8.0
MCULE-5843881524-0-3	His250	Leu65, Val73, Trp93, Lys211, His250#	His250 *	Phe70, Ser217, Gly219, Asn220	−6.9
MCULE-7157846117-0-1	Gln123	Leu65, Val73, Trp93 *, His250	-	Met67, His189, Lys211, Gly219, Asn220	−7.1
MCULE-8777613195-0-12	His120, His122, His189, Cys208	Trp93	-	Zn1, Zn2, Phe70, Val73, Gln123, Asp124, Lys211, Gly219, Asn220, His250	−8.9

* Two bonds. # Three bonds.

**Table 3:** ADMET properties of the selected molecules deduced by SwissADME.

	MCULE-1996250788-0-2	MCULE-8777613195-0-12	MCULE-2896881895-0-14	MCULE-5843881524-0-3	MCULE-4937132985-0-1	MCULE-7157846117-0-1
**Physicochemical properties**						
Formula	C_22_H_16_N_10_	C_20_H_14_N_6_	C_20_H_21_N_2_OP	C_17_H_12_BrN_5_S	C_21_H_21_N_3_O_2_	C_20_H_21_N_3_O_3_
Molecular wt (g/mol)	420.43	338.37	336.37	398.28	347.41	351.40
Rotatable bonds	4	0	7	3	3	4
H-bond acceptors	7	4	1	3	3	5
H-bond donors	0	2	2	2	1	0
Molar refractivity	119.12	110.71	103.06	105.30	104.91	102.74
TPSA (Å^2^)	103.33	78.12	50.94	103.90	56.15	56.71
**Lipophilicity**						
Log Po/w (iLOGP)	3.50	2.49	2.81	2.60	3.55	3.51
Log Po/w (XLOGP3)	4.36	3.43	4.49	4.26	4.36	3.34
Log Po/w (WLOGP)	3.11	0.38	4.43	3.49	3.34	2.52
Log Po/w (MLOGP)	4.37	2.76	3.21	3.32	3.69	1.51
Log Po/w (SILICOS-IT)	0.74	0.26	3.28	4.99	3.75	2.98
Consensus Log Po/w	3.21	1.86	3.64	3.73	3.74	2.77
**Water solubility**						
Log S (ESOL)	−5.62	−4.44	−4.85	−5.32	−5.03	−4.31
Solubility (mol/l)	2.38 × 10^−6^	3.63 × 10^−5^	1.42 × 10^−5^	4.79 × 10^−6^	9.41 × 10^−6^	4.85 × 10^−5^
Class	Moderate	Moderate	Moderate	Moderate	Moderate	Moderate
Log S (Ali)	−6.25	−4.75	−5.28	−6.15	−5.25	−4.21
Solubility (mol/l)	5.68 × 10^−7^	1.77 × 10^−5^	5.25 × 10^−6^	7.02 × 10^−7^	5.56 × 10^−6^	6.19 × 10^−5^
Class	Poor	Moderate	Moderate	Poor	Moderate	Moderate
Log S (SILICOS-IT)	−7.09	−4.53	−8.33	−7.41	−6.74	−6.02
Solubility (mol/l)	8.13 × 10^−8^	2.99 × 10^−5^	4.67 × 10−^9^	3.85 × 10^−8^	1.83 × 10^−7^	9.64 × 10^−7^
Class	Poor	Moderate	Poor	Poor	Poor	Poor
**Pharmacokinetics**						
GI absorption	High	High	High	High	High	High
BBB permeant	No	No	Yes	No	Yes	Yes
P-gp substrate	Yes	Yes	No	No	No	No
CYP1A2 inhibitor	No	No	Yes	Yes	Yes	Yes
CYP2C19 inhibitor	No	Yes	Yes	Yes	Yes	Yes
CYP2C9 inhibitor	Yes	Yes	Yes	Yes	Yes	Yes
CYP2D6 inhibitor	No	No	Yes	No	No	Yes
CYP3A4 inhibitor	No	No	Yes	Yes	Yes	Yes
Log *K*_p_ (skin permeation)	−5.77 cm/s	−5.93 cm/s	−5.16 cm/s	−5.70 cm/s	−5.32 cm/s	−6.07 cm/s
**Druglikeness**						
Lipinski (#violations)	Yes; 1	Yes; 0	Yes; 0	Yes; 0	Yes; 0	Yes; 0
Ghose	Yes	Yes	Yes	Yes	Yes	Yes
Veber	Yes	Yes	Yes	Yes	Yes	Yes
Egan	Yes	Yes	Yes	Yes	Yes	Yes
Muegge	Yes	Yes	Yes	Yes	Yes	Yes
Bioavailability score	0.55	0.55	0.55	0.55	0.55	0.55
**Medicinal Chemistry**						
PAINS	0 alert	0 alert	0 alert	0 alert	0 alert	0 alert
Brenk	0 alert	0 alert	1 alert	1 alert	0 alert	0 alert
Leadlikeness (#violations)	No; 2	Yes	No; 1	No; 2	No; 1	No; 1
Synthetic accessibility	3.46	4.35	4.19	3.56	3.75	3.29

**Table 4:** Enzyme kinetics parameters of NDM-1 in the presence of MCULE-8777613195-0-12.

Antibiotics	*K*_m_ (µM)	*k*_cat_ (s^−1^)	*k*_cat_/*K*_m_ (µM^−1^ s^−1^)
***NDM-1 alone***			
nitrocefin	27.1 ± 2.1	271.2 ± 12.6	10.01 ± 0.92
ampicillin	99.4 ± 7.6	392.1 ± 25.7	3.94 ± 0.14
cefotaxime	61.6 ± 5.8	412.6 ± 28.1	6.70 ± 0.27
imipenem	83.9 ± 6.7	700.3 ± 18.7	8.34 ± 0.64
meropenem	58.3 ± 5.2	301.4 ± 11.9	5.17 ± 0.33
***NDM-1 + MCULE-8777613195-0-12***			
nitrocefin	48.9 ± 4.2	112.3 ± 6.2	2.30 ± 0.11
ampicillin	100.5 ± 8.3	158.7 ± 7.6	1.58 ± 0.07
cefotaxime	124.5 ± 9.9	126.4 ± 5.1	1.02 ± 0.07
imipenem	98.7 ± 6.1	156.2 ± 6.9	1.58 ± 0.06
meropenem	101.7 ± 7.3	138.4 ± 8.0	1.36 ± 0.04
***NDM-1 + D-captopril (control)***			
nitrocefin	78.6 ± 4.4	162.8 ± 16.3	2.07 ± 0.15

## Data Availability

Not applicable.

## Supplementary Materials

The following are available online at https://www.mdpi.com/article/10.3390/molecules27185930/s1, Supplementary Figure S1: Validation of molecular docking protocol by redocking the ligand (present in the X-ray structure) to the active site of NDM-1, and computing the RMSD (root mean square deviation) by comparing docked pose of ligand to that of the crystal structure pose. Supplementary Figure S2: (A) Binding of different ligands to the substrate binding site of NDM-1. Binding pose and interaction of NDM-1 with (B) MCULE-1996250788-0-2; (C) binding of MCULE-2896881895-0-14; (D) MCULE-4937132985-0-1; (E) MCULE-5843881524-0-3; (F) MCULE-7157846117-0-1.
