# Supplementary material for: Identification of a Potential Inhibitor (MCULE-8777613195-0-12) of New Delhi Metallo-β-Lactamase-1 (NDM-1) Using In Silico and In Vitro Approaches
Source: Molecules. 2022 Sep 13;27(18):5930. doi: 10.3390/molecules27185930 (PMC9504514; doi:10.3390/molecules27185930)
Supplement: Supplementary file 1 [file molecules-27-05930-s001.zip › molecules-1837198-supplementary.pdf]

Supplementary Materials

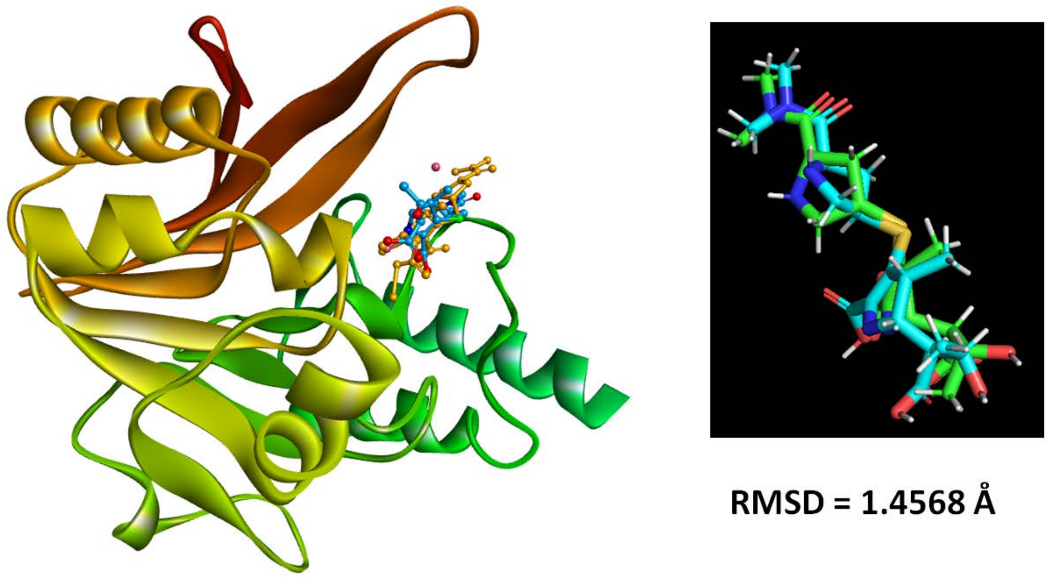

Supplementary Figure S1: Validation of molecular docking protocol by redocking the ligand (present in the X-ray structure) to the active site of NDM-1, and computing the RMSD (root mean square deviation) by comparing docked pose of ligand to that of the crystal structure pose.

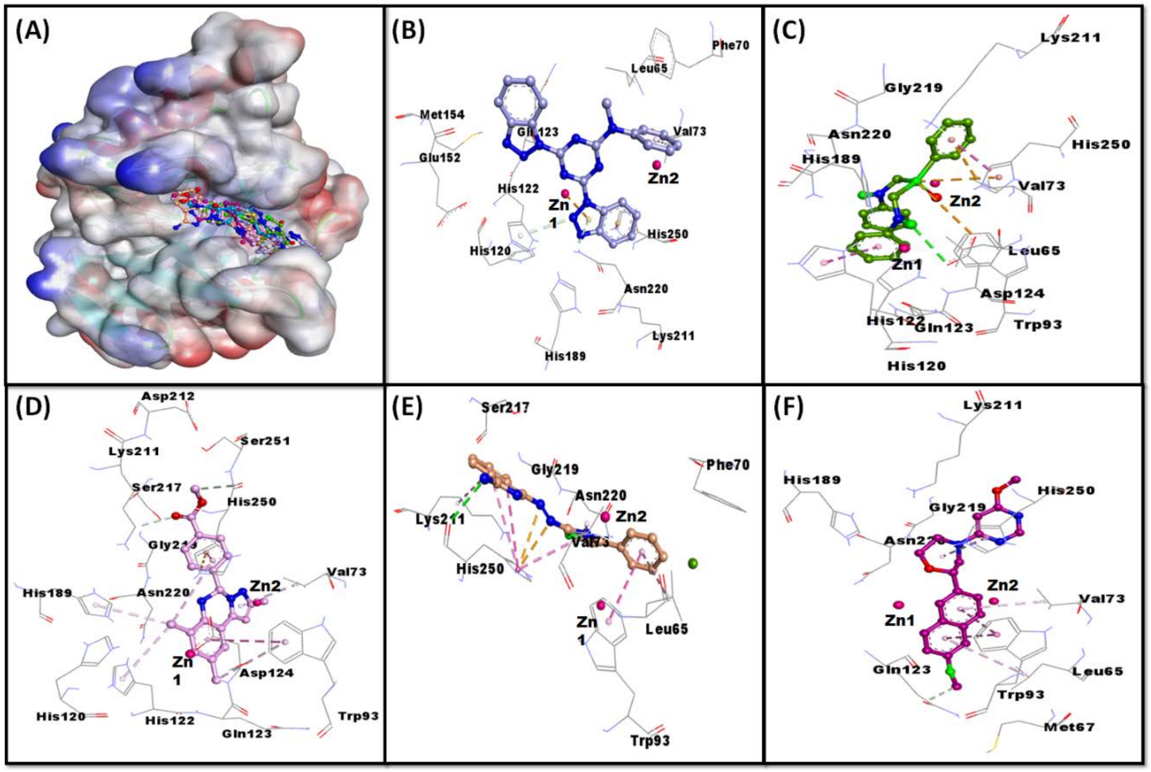

Supplementary Figure S2: (**A**) Binding of different ligands to the substrate binding site of NDM-1. Binding pose and interaction of NDM-1 with (**B**) MCULE-1996250788-0-2; (**C**) binding of MCULE-2896881895-0-14; (**D**) MCULE-4937132985-0-1; (**E**) MCULE-5843881524-0-3; (**F**) MCULE-7157846117-0-1
